# Comparison of host immune responses to LPS in human using an immune profiling panel, *in vivo* endotoxemia *versus ex vivo* stimulation

**DOI:** 10.1038/s41598-020-66695-2

**Published:** 2020-06-18

**Authors:** Dina M. Tawfik, Jacqueline M. Lankelma, Laurence Vachot, Elisabeth Cerrato, Alexandre Pachot, W. Joost Wiersinga, Julien Textoris

**Affiliations:** 1 EA7426 “Pathophysiology of Injury-Induced Immunosuppression”, PI3, Université Claude Bernard Lyon-1 Hospices Civils de Lyon - bioMérieux, Lyon, France; 2 Open Innovation and Partnerships (OIP), bioMérieux S.A, Lyon, France; 3 Department of Medical Microbiology and Infection Control, Amsterdam UMC, Location Vrije Universiteit Amsterdam, Amsterdam UMC, Amsterdam, The Netherlands; 4 Center for Experimental and Molecular Medicine, Amsterdam UMC, location Academic Medical Center, University of Amsterdam, Amsterdam, The Netherlands; 5 Division of Infectious Diseases, Department of Medicine, Amsterdam UMC, location Academic Medical Center, University of Amsterdam, Amsterdam, The Netherlands; 6 Anaesthesia and Critical Care Medicine Department, Hospices Civils de Lyon, Edouard Herriot Hospital, Lyon, France

**Keywords:** Immunogenetics, Infection, Inflammation, Innate immune cells, Innate immunity, Lymphocytes, Translational immunology, Biomarkers, Immunological disorders

## Abstract

Patients that suffer from sepsis exhibit an early hyper-inflammatory immune response which can lead to organ failure and death. In our study, we assessed the immune modulation in the human *in vivo* endotoxemia model and compared it to *ex vivo* LPS stimulation using 38 transcriptomic markers. Blood was collected before and after 4 hours of LPS challenge and tested with the Immune Profiling Panel (IPP) using the FilmArray system. The use of IPP showed that markers from the innate immunity dominated the response to LPS *in vivo*, mainly markers related to monocytes and neutrophils. Comparing the two models, *in vivo* and *ex vivo*, revealed that most of the markers were modulated in a similar pattern (68%). Some cytokine markers such as *TNF*, *IFN-γ* and *IL-1β* were under-expressed *ex vivo* compared to *in vivo*. T-cell markers were either unchanged or up-modulated *ex vivo*, compared to a down-modulation *in vivo*. Interestingly, markers related to neutrophils were expressed in opposite directions, which might be due to the presence of cell recruitment and feedback loops *in vivo*. The IPP tool was able to capture the early immune response in both the human *in vivo* endotoxemia model, a translational model mimicking the immune response observed in septic patients.

## Introduction

The host immune response in sepsis is currently known as an initial phase of a hyper-inflammatory response, that is concomitantly met with an anti-inflammatory response to restore homeostasis^1^. In the Intensive Care Unit (ICU), mortality attributable to sepsis can reach up to 45%^2^ due to organ failure – a consequence of a dysregulated host response including inflammatory cytokine storm– and/or acquiring secondary infections - with concurrent sepsis-induced immunoparalysis^1^. The major challenge in managing septic patients is the high heterogeneity in host responses due to inter-individual variability, the pathogen or source of infection and varying responses to treatment, which lead to different immune trajectories and outcomes^3,4^. Many clinical trials have been conducted, and are currently ongoing, to test the efficacy of several immune-directed therapies to improve patient outcomes. For instance, anti-inflammatory agents such as Interleukin-1 Receptor antagonist (*IL-1Ra*)^5^ and immune stimulatory agents such as interleukin-7 (*IL-7*)^6^ and interferon-gamma (*IFN-γ*)^7–9^ and others with promising results are currently under evaluation. Nonetheless, it is not yet feasible to easily identify and stratify patients with different immune profiles in the ICU that would benefit from such treatment in day-to-day clinical practice^10^. The availability of an immune profiling tool based on immune biomarkers can help determine the superseding immune dysfunction (hyper inflammation or immune suppression) in septic patients. Such tool can be used in personalized medicine to help stratify and assign patients that are likely to benefit from a targeted immunomodulatory agent and monitor their response to treatment^7,11^.

Lipopolysaccharide (LPS) is a highly antigenic molecule that is derived from Gram-negative bacteria and activates a plethora of inflammatory cascades via Toll-like receptor-4 (TLR-4)^12^. LPS stimulation can mimic the initial acute inflammatory response to sepsis, both *in vivo* and *ex vivo*. The human *in vivo* endotoxemia model is a well-established model that can capture the host response from half an hour after the challenge and  up to 20 days^13^. *In vivo* endotoxemia subjects experience transient symptoms reminiscent of systemic infection in sepsis that include fever, chills, headaches and nausea. These symptoms are accompanied by an increase in blood pressure, heart rate, as well as neutrophilia, lymphocytopenia and monocyte anergy^14,15^. Other widely used model in immunological studies is the *ex vivo* LPS stimulation of whole blood. The use of *ex vivo* models helped unravel many underlying mechanisms of the inflammatory response, and it can also be utilized as a standalone test of the immune function^16^. The safety, non-invasiveness and flexibility of testing different stimulants make the *ex vivo* model easy-to-use and appealing to researchers. The *ex vivo* model has also proved valuable in the diagnostic field, where functional assays such as TNF-α release post-LPS stimulation has been used to test the immune competency of Peripheral Blood Mononuclear Cells (PBMCs) of patients^10^. Such well-established model can help untangle the complexity of host responses at the transcriptomic and proteomic levels. The conditions of the *ex vivo* model are easier to control and replicate, while *in vivo*, the model better mimics the human or patients’ host response to inflammation, as it allows the occurrence of positive and negative feedback loops, in addition to recruitment and mobilization of various subsets of immune cells^15,17^. However, setting up an *in vivo* human endotoxemia model is met with several barriers such as the rules and regulations of each country, the need for screening and careful recruitment of volunteers (to minimize heterogeneity), the potential risks for healthy volunteers, and the continuous monitoring directly afterwards.

In this study, we used a tool we recently described: the Immune Profiling Panel (IPP), a multiplex molecular tool using the FilmArray system^18^, to assess the host response to LPS stimulation, using 38 immune-related transcriptome markers *in vivo*. The panel of markers targeted several immune functions, cell surface markers and the two arms of the immune response including inflammatory and anti-inflammatory responses. We compared the gene modulation *in vivo* versus *ex vivo* LPS stimulation of whole blood samples from healthy volunteers, in order to better understand the ability and limitations of each model in capturing  the complexity of the host response to endotoxemia.

## Material and Methods

### Healthy volunteers

Eight healthy, non-smoking, Caucasian male subjects (aged 18–25 years) from the Netherlands were examined, screened and recruited for studying the *in vivo* human endotoxemia model^14^. The volunteers received an LPS bolus infusion (*E. coli* O113 Reference Endotoxin, National Institutes of Health, Bethesda, Maryland, USA) at a dose of 2 ng/kg bodyweight. Whole blood (2.5 mL) was collected in PAXgene tubes (Pre-Analytix, Hilden, Germany) at T0 (before LPS injection) and T4 (4 hours after LPS injection). The PAXgene tubes were inverted several times and incubated for 2 hours at room temperature according to the manufacturer’s recommendation, and stored at -80 °C until the transcriptomic testing. The blood cell counts were performed using fully automated hematology analyser XN-9000 (Sysmex, Etten-Leur, The Netherlands). The study was approved by the institutional ethics and research board (Amsterdam University Medical Centers, location AMC; Netherlands Trial Registration number NL4425 (NTR4549); ClinicalTrials.gov identifier NCT02127749). All subjects gave a written informed consent and research was conducted in accordance with the declaration of Helsinki.

For the *ex vivo* LPS stimulation, whole blood was collected in heparin tubes from 8 healthy volunteers, 6 males and 2 females (aged 21–55 years) obtained from the EFS (Etablissement Français du Sang, French blood bank, Lyon, France). One milliliter of heparinized blood was distributed into pre-warmed TruCulture tubes (Myriad Rbm, Austin, TX, USA) containing 2 ml of medium alone (Nul) or medium with LPS (*E. coli* O55:B5; 100 ng/mL). The TruCulture tube system was selected for the *ex vivo* model as it is an easy-to-use culture tube supplied with a standardized culture medium and LPS, thus, designed for use as standardized diagnostic tests (currently available for Research Use Only)^19,20^. The samples were incubated in a dry block at 37 °C for 4 hours and directly tested in the IPP prototype. Informed consents from the blood donors were obtained and their personal data were anonymized at the time of blood donation and before reaching our laboratory according to EFS institutional regulations.

### Transcriptome analysis

Stimulated whole blood samples (PAXgene *in vivo* samples and TruCulture stimulated *ex vivo* samples) were tested with IPP prototype^18^ according to manufacturer’s instructions. Briefly, the pouches were hydrated with the hydration solution supplied with the kit. A 100 μL of either PAXgene mix or TruCulture mix were mixed with approximately 800 µL of the lysis buffer provided with the kit and directly injected into the pouch and ran on FilmArray 2.0 and FilmArray Torch instruments (BioFire, Inc., Salt Lake City, UT, USA). Results were delivered in less than 1 hour and the normalized expression values of markers were computed^18^.

### Statistical analyses

Normalized expression values were analyzed using hierarchical clustering (Euclidean distance) and scaled to demonstrate the differential modulations of genes in a heatmap plot. Normalized data used in Principal Component Analyses (PCA) were scaled and centered. Missing values in the biological parameters and cells count were imputed using missMDA package^21^, where a PCA model was used to estimate the missing values to perform the PCA- Biplot. Normalized expression levels of genes were expressed as median and interquartile ranges (IQR) box and whisker plots. Samples were compared before and after LPS stimulation using Mann-Whitney U test for unpaired samples (*in vivo* against *ex vivo* model), while paired Wilcoxon signed-rank test was used for paired samples (*in vivo* model T4 against T0. P-values were adjusted using FDR (Benjamini-Hochberg False Detection Rate) to correct for multiple testing^22^. The level of significance was set at 5% two-sided tests. Statistical analyses were performed and computed using R software version 3.5.1(R core Team, Vienna, Austria)^23^.

## Results

### Gene modulation in the *in vivo* human endotoxemia model

**Figure 1:**
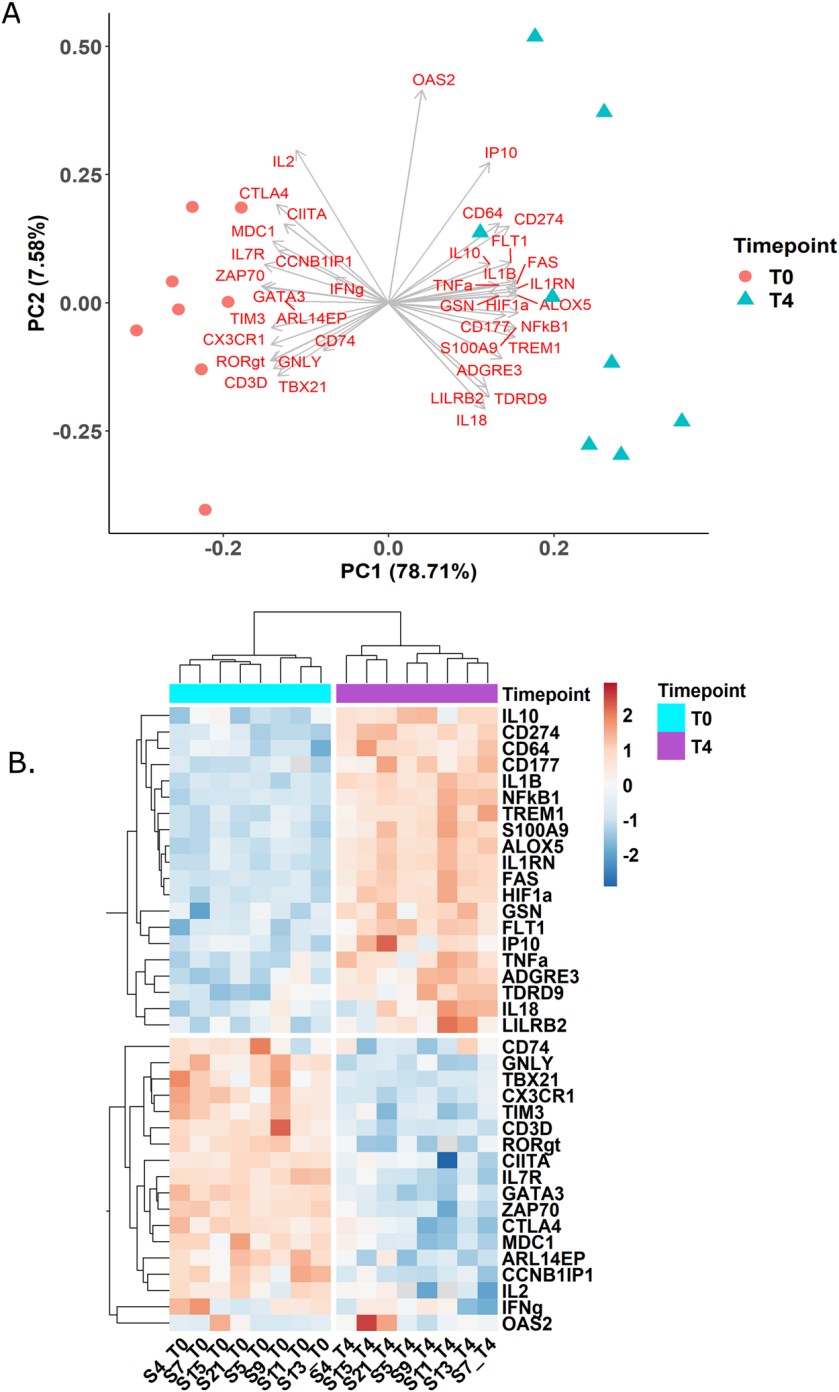
Gene modulations captured by IPP in the  human *in vivo* endotoxemia model. (**A**) Principal component (PC) analysis of the samples before and after stimulation, where the colors differentiate the time points T0 (just before receiving the LPS bolus infusion) and T4 (4 hours after LPS injection). (**B**) Heatmap showing the hierarchical clustering of the samples (T0 and T4) and genes using Euclidean distance. Normalized gene expression values are color-coded from blue (down-modulation) to red (up-modulation).

The IPP tool captured the host immune response in whole blood at the transcriptomic level 4 hours after LPS injection. The PCA illustrates that LPS stimulation drives the variability seen in the dataset (Fig. 1A). All the unstimulated individuals (T0) are grouped on the left-hand side of the plot, and the stimulated individuals (T4) are sparsely grouped in the opposite direction. Principal component-1 (x-axis) explains 78.7% of the variability in the dataset and separates the two time points (before and after the LPS challenge).

In the upper panel of the heatmap (Fig. 1B), an up-modulation can be observed for 20 genes at T4. Markers from the innate immune response dominated the up-modulated genes, which included inflammatory mediators such as *NFkB1* (transcription factor involved in inflammatory response) and *ALOX5* (involved in leukotrienes synthesis from arachidonic acid associated with inflammation), early pro-inflammatory cytokines *TNF* and *IL-1*β, anti-inflammatory cytokines *IL-10* and *IL1RN*, and neutrophil cell surface markers *CD177* (neutrophil-specific marker)*, CD64*, *TREM1*, and *S100A9*. The latter three are markers expressed on the surface of both neutrophil and monocyte cells, which are  the first line of cells that respond to the LPS challenge.

The lower panel highlights 18 genes that were down-modulated at T4. These included monocyte and T-cell surface markers, in addition to factors involved in the activation and differentiation of T-cells. Monocyte surface markers *CX3CR1* (fractalkine receptor that has a major role in monocytes survival), *CD74* (the invariant chain of HLA-DR) and *CIITA* (major histocompatibility complex class II transactivator) were all down-modulated. Also, a decreased expression of T-cell markers including *IL7R* (associated with lymphocyte development), *TBX21* (Th_1_ cell-specific transcription factor), and *ZAP70* (part of T cell receptor, and plays a critical role in T-cell signaling) indicating a compromised functionality of lymphocytes. A decrease in *CD3D* (encodes T-cell surface glycoprotein CD3 delta chain) and *GATA3* (regulator of T-cell development) expression was also captured by the IPP tool which might be related to a decline in the lymphocyte count.

### Biological parameters and immune cell modulation observed in the *in vivo* endotoxemia model

The kinetic changes in the absolute count of immune cells before and after the LPS stimulation were recorded in our prior study^14^. A marked decrease was observed in monocyte, eosinophil and lymphocyte populations while a 2-fold increase in the neutrophil count was detected after 4 hours of LPS stimulation (Supplementary Fig. S1).

**Figure 2:**
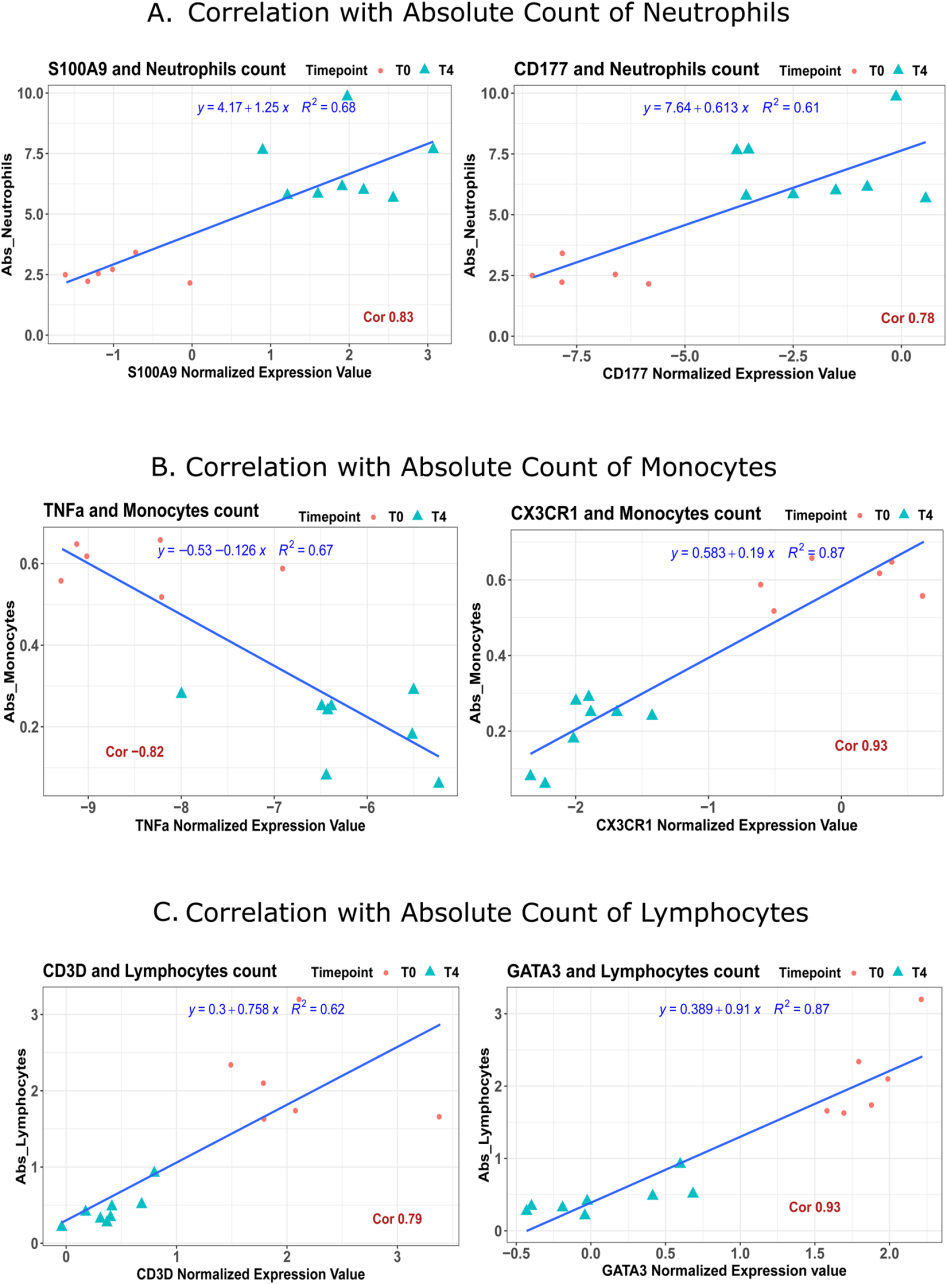
Pearson correlations of IPP markers with the absolute count of immune cells. Six IPP markers were correlated with the absolute count of immune cells at T0 (red cirlces) and T4 (blue triangles), with a strong correlations above 0.7 (Pearson coefficient). All correlations were statistically significant p < 0.001. A positive correlations indicates that the normalized expression of IPP markers increases as the count increases. (**A**) correlations between S100A9 and CD177 expression with neutrophils count. (**B**) Correlation between TNF and CX3CR1, and monocytes count. (**C**) Correlation between CD3D and GATA3, and lymphocytes count.

The biological parameters measured for the same volunteers were analyzed using PCA to identify and evaluate the contribution of each variable to the separation of T0 and T4. The highest contributing variables to the observed responses were the absolute count of monocytes and lymphocytes, as well as coagulation factors II and X, prothrombin time (PT), Plasminogen activator inhibitor-1 (PAI-1), inflammatory cytokines: *IL6*, *IL8*, *TNF-α* and anti-inflammatory cytokine *IL10* (Supplementary Fig. S2). The correlations of various markers that are biologically related to immune cell counts are illustrated in Fig. 2*. S100A9* and *CD177* had a strong correlation (Pearson coefficient > 0.7) with neutrophils count. Monocytes count was also strongly correlated; positively with *CX3CR1* and negatively with *TNF* expression (Pearson coefficient > 0.8). As for the lymphocytes count, it was strongly correlated with *CD3D* and *GATA3* (Pearson coefficient > 0.7). The rest of the correlations between the biological markers and cell counts with IPP markers were above 0.7 (Pearson coefficient), an indicator for a strong correlation, are shown in Supplementary Fig. S3.

To assess the impact of immune cell count changes on gene expression, we adjusted the expression level of each marker to the absolute number of the corresponding immune cell. Most of the markers remained differentially expressed between T0 and T4, with the exception of *CD64*, *GATA3* and *CD3D* (Fig. S4). In the case of *CD177* and *CD74*, we observed a clear differential expression that did not reach statistical significance, which might be due to the limited number of samples.

### Comparison of the *in vivo* Human Endotoxemia Model with the *ex vivo* LPS Stimulation in TruCulture System

**Figure 3:**
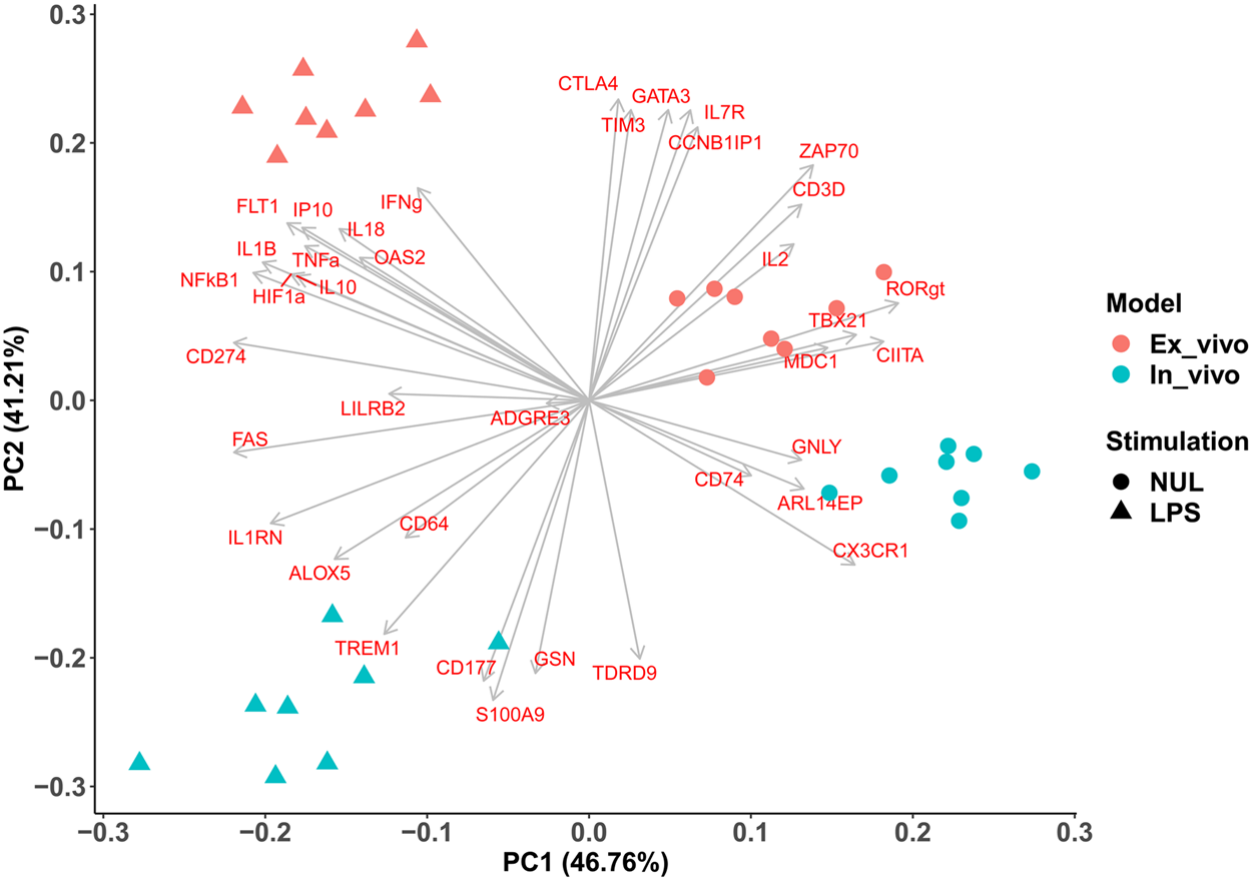
Comparison between *in vivo* and *ex vivo* LPS stimulation using IPP. PC analysis was used to compare the difference of expression between the human *in vivo* endotoxemia model (blue) and the *ex vivo* stimulation model (red) before (NUL, circles) and after the LPS challenge (LPS, triangles). The contribution of each gene to the plot is represented as arrows.

IPP markers were used to study the difference between the *in vivo* endotoxemia model and LPS blood stimulation *ex vivo*. The majority of the IPP markers (68%) showed a similar pattern of expression in both models post-LPS stimulation. PCA analysis was performed on the entire dataset (Fig. 3), and it can be observed that the unstimulated (NUL) *ex vivo* samples clustered closely to the *in vivo* control samples on the first PCA axis, while stimulated samples (T4) were grouped on the other side of the same axis (PC-1). Principal component-1 (x-axis) represents 47% of the dataset variability and separates the LPS response after 4 hours from unstimulated samples with an equal distance. Of particular interest is PC-2 (y-axis) in this analysis, which separates the two LPS responses according to the type of model whether *ex vivo* or *in vivo*, explaining 41.2% of the variability of the whole dataset (Fig. 3). Interestingly, Fig. 3 shows that the direction of variance is opposite in the two models. The genes that mostly contributed to this difference along PC-2 axis were *TDRD9*, *GSN*, *S100A9*, *CD177*, *TREM1*, *CD64*, *ALOX5, ADGRE3* and *IL1RN* (up-regulated genes *in vivo*), and *CTLA4*, *TIM3*, *GATA3*, *IL7R*, *CCNB1IP1* and *ZAP70* genes (down-regulated *in vivo*).

**Figure 4:**
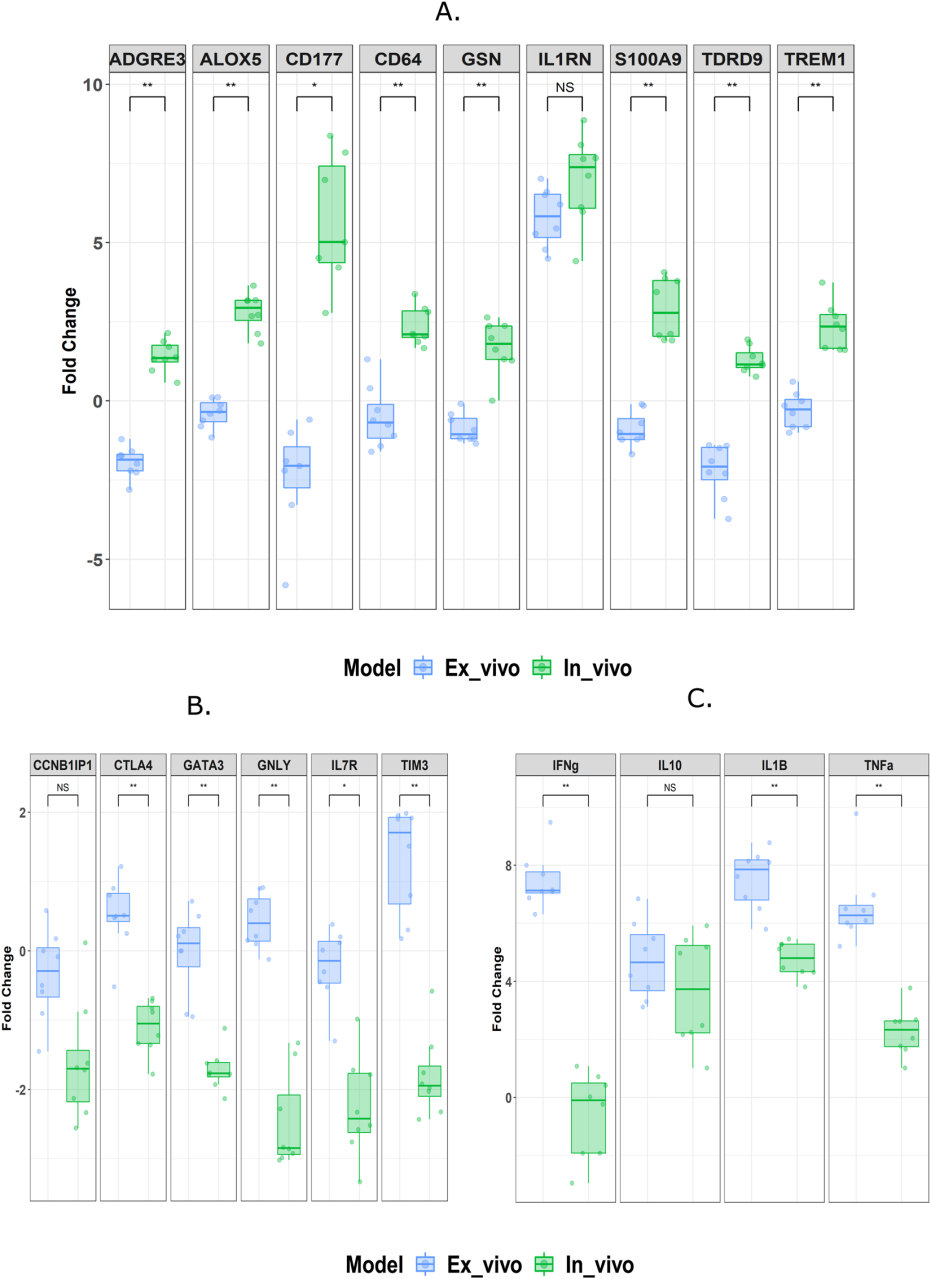
Boxplots showing the fold change of IPP markers observed between the *in vivo* and the *ex vivo* models after LPS stimulation. On the y-axis is the fold change of both models (normalized expression in the stimulated condition (LPS) minus the unstimulated condition) presented as boxplots, *in vitro* stimulation (blue) and *in vivo* endotoxemia (green). (**A**) Fold change of markers associated with the early innate immune response. (**B**) Fold change of markers associated with the adaptive immune response. (**C**) Fold change of markers associated with inflammatory and anti-inflammatory cytokines. Fold changes were compared using Mann-Whitney U test and P values were adjusted using FDR (Benjamini-Hochberg), where NS: p > 0.05, *p < 0.05, **p < 0.01 and ***p < 0.001.

We compared the markers that showed an opposite and different magnitude of expression patterns between the two models, by subtracting the unstimulated expression from the LPS stimulation for each marker (Fig. 4). The comparison revealed that the expression of *ADGRE3*, *ALOX5*, *CD177*, *CD64*, *GSN, S100A9* and *TREM1* were up-modulated only in the *in vivo* model, which are chiefly associated with the early innate immune response (Fig. 4A). In contrast, some genes were down-modulated *in vivo* only and were mainly related to the adaptive immune response (*IL7R*, *CTLA4*, *GATA3*, *TIM3*, and *GNLY)* (Fig. 4B). Interestingly, some markers had different magnitudes of expression in the two models, such as *IFN-γ*, *IL-1β*, and *TNF*, and were strongly up-regulated *ex vivo* compared to the *in vivo* model (Fig. 4C). The comparison between the two models highlighted that the majority of the IPP markers were expressed in a similar pattern, while others, interestingly, were mostly modulated *in vivo* only, and clearly discriminated the two models.

## Discussion

The *in vivo* human endotoxemia model is a translational model that can be used to mimic the acute inflammatory profile reported in septic patients^15^. Once the volunteers are challenged with LPS, the innate immune response is triggered initiating several pro-inflammatory cascades and the secretion of inflammatory cytokines, quickly followed by an increase in neutrophil count^24^. The peak of neutrophilia, mono-, eosino- and lympho-cytopenia occurred at 2–4 hours after the endotoxin challenge *in vivo*^25–28^. This initial systemic inflammatory response is concomitantly met with an anti-inflammatory response to restore homeostasis. In sepsis, this immune response can become dysregulated causing detrimental consequences. The use of the Immune Profiling Panel (IPP) helped capture the gene modulations observed in the *in vivo* endotoxemia model. An increase in neutrophil-associated markers (*S100A9, TREM1* and neutrophil specific marker *CD177*^29^) reflects the increased recruitment and activation of neutrophils, while the increased expression of *CD64* might indicate an increase in neutrophil count. A down-modulation in monocyte surface markers (*CX3CR1, CIITA* and *CD74*, a surrogate marker for HLA-DR^30^) may indicate monocyte deactivation. The down-modulation of the adaptive immune markers (*GNLY, TBX21, RORγt, CTLA4*) highlights an alteration in lymphocyte function, signaling and the activation of T_h1_, T_h17_ and T_reg_ cells. When *CD3D* and *GATA3* were adjusted to the lymphocyte count they seem to be non-differentially expressed indicating that their expression might mainly reflect lymphocytopenia. An overall increase was observed in both pro- and anti-inflammatory cytokines expression such as *TNF, IL-1β, IFN-γ, IL10* and *IL1RN* after stimulation (Fig. 1B). Each of these modulations pinpoints to a functional alteration, that resembles the acute inflammatory phase observed in septic patients^31^. These results underscore future possibilities of using novel molecular tools such as IPP at the bedside in the ICU to help characterize the immune profile of patients at risk, enabling personalized medicine based on immune markers.

In the second part of our study, we compared the same 38 IPP markers with the *ex vivo* LPS stimulation of whole blood after 4 hours. Most of the markers (~70%) were expressed in a similar pattern and direction with minor non-significant differences in magnitude (data not shown). This slight difference in magnitude between the two models might be due to the use of different healthy volunteers or the type and concentration of LPS. Recently, two markers S100A9 and TREM1 which are included in our panel were identified as LPS dose-dependent, where their expression increased with an increase in the LPS dose^32^. Curiously, some markers such as *TNF* were highly expressed *ex vivo* compared to the *in vivo* model, while *IL10* had the same expression level in both models (Fig. 4C). These results differ from the protein data reported by Dorresteijn *et al*., where *TNF* and *IL10* levels were higher *in vivo* compared to *ex vivo*^17^. In our data, *IL-1β* and *IFN-γ* were highly expressed *ex vivo* compared to *in vivo* after 4 hours of stimulation, which corresponds with the findings of Dorresteijn *et al*. at the protein level 24 hours post-stimulation^17^. Nonetheless, the difference in timing regimens and the nature of markers (transcriptome versus protein) might also contribute to the differences reported above. Sometimes, the local production of cytokines in tissues exert a “spillover effect”, where the tissues can pour cytokines into peripheral blood, thus increasing the serum levels^33^. Although transcriptomic tests are more sensitive than protein detection, the post-transcriptional regulation mechanisms can vary across tissues, and the detection of mRNA does not necessarily imply its translation^17^. The stability of transcripts can also affect their detection in blood, for instance, *IL1RN* mRNA tends to remain effective and detectable in blood after 4 hours compared to the mRNA of *IL-1β* which decays rapidly (T_1/2_ ~1.8 hours)^34^. All the previous points should be considered when extrapolating results from the *ex vivo* model to the *in vivo* model.

Interestingly, some markers showed an opposite expression pattern between the two models. Most of these markers were related to neutrophils such as *CD177* as well as *CD64*, *S100A9* and *TREM1* that are highly expressed on the surface of both monocytes and neutrophils. This phenomenon might be observed *in vivo* due to the direct stimulation of circulating neutrophils by LPS, and the recruitment of new neutrophils from the bone marrow^25,29^. Besides, the *ex vivo* model is mainly dependent on peripheral blood, while *in vivo* has both tissue and blood compartments, actively contributing to the detected response. However, this is not the case for the *ex vivo* model that lacks feedback loops, cell recruitment and communication with the tissue compartment. Our data also showed that the down-modulation in the adaptive immune response markers (*CTLA4, GATA3, GNLY, IL7 and TIM3*) are linked to the function and activation of T-cells in the *in vivo* model. This interesting observation might be due to the reduced capacity of antigen-presenting cells to activate the T-cells post-LPS challenge^35^.

One of our limitations is the use of different *E. coli* LPS serotypes and concentration, which could contribute to the difference in the host response observed between the two models. However, we wanted to assess the ability of *ex vivo* and *in vivo* models to capture the complexity of the host response to LPS and understand their limitations in a standardized manner. This will help provide insights and data about the tool’s ability to capture such modulations which will make it comparable to future studies and tool users. Another limitation was the use of a mixed-gender population for the *ex vivo* model in comparison to an all-male population *in vivo*, which might influence the differences observed in immune response between the two models. Wegner *et al*. observed a higher pro-inflammatory cytokine response to LPS in females compared to males, which was significant only *in vivo* but not *ex vivo*^36^. Nonetheless, we performed a preliminary analysis on the healthy volunteers population (n = 180) in the REALISM project^37^ and so far we didn’t observe a significant gender-related difference for the transcriptomic markers included in the IPP tool (unpublished data).

To the best of our knowledge, this is the first study to report a direct comparison between the *in vivo* and *ex vivo* models after only 4 hours of LPS stimulation using transcriptomic immune markers. This study underscores that caution must be taken when interpreting and extrapolating the results of certain markers from the *ex vivo* model. Our study also highlights how employing an immune profiling panel can capture and describe the host immune responses *in vivo* after 4 hours of LPS stimulation. The use of the *in vivo* human endotoxemia model showed gene modulations and alterations similar to those observed in the early inflammatory response associated with sepsis syndrome and other critical conditions in the ICU. However, in sepsis, a complex dysregulation in the immune response can occur such as the early onset of immunosuppression making it hard to predict the disease trajectory or outcomes. More than 100 clinical trials were conducted on novel immunotherapies in sepsis, those trials mainly targeted the modulation of the systemic inflammatory response in patients with acute clinical manifestations. However, almost none of these trials have resulted in new treatments available in the market. Those clinical trials might have failed as all patients were treated in the same manner, and few patient stratification approaches were adopted^11,38^. Septic patients have heterogeneous responses and a patient stratification strategy based on a panel of immune biomarkers can help identify an individual patient’s dysfunction^11^. An immune profiling tool can guide the use of targeted immunotherapies and contribute to the success of future clinical trials. Testing IPP in human *in vivo* endotoxemia model showed the potential value of such tool to determine the immune status of critically-ill patients. The immune profiling panel has 5-mins only hands-on, immune markers are measured directly from whole blood and results can be achieved in less than an hour. The ease and rapid turnaround time of IPP suggest its future use at the bedside compared to the currently available techniques that offer limited information about the host response and require long and complicated technical procedures. Our next step is to evaluate the capability and the predictive performances of the IPP markers in a dedicated cohort of critically-ill patients to stratify patients at high risk of stratify patients at high risk of deterioration and enable the use of new immunotherapies and personalized medicine.

## Supplementary information

Supplementary information.
