# Supplementary material for: Comparison of host immune responses to LPS in human using an immune profiling panel, in vivo endotoxemia versus ex vivo stimulation
Source: Sci Rep. 2020 Jun 18;10:9918. doi: 10.1038/s41598-020-66695-2 (PMC7303162; doi:10.1038/s41598-020-66695-2)

Supplementary Information

Comparison of host immune responses to LPS in human using an immune profiling panel, *in vivo* endotoxemia *versus ex vivo* stimulation

Dina M. Tawfik, Jacqueline M. Lankelma, Laurence Vachot, Elisabeth Cerrato, Alexandre Pachot, W. Joost Wiersinga, Julien Textoris

Table S1 The Immune profiling panel (IPP). The table includes the name of each marker in the IPP tool with the transcripts accession number.

Gene	Name	Accession number		Gene	Name	Accession number
ADGRE3	Adhesion G protein-coupled receptor E3	NM_032571		***IL10***	Interleukin 10	NM_000572
ALOX5	Arachidonate 5-lipoxygenase	NM_001256154		***IL18***	Interleukin 18	NM_001562<br>NM_001243211
ARL14EP	Ribosylation factor like GTPase 14 effector protein	NM_152316		***IL1B***	Interleukin 1 beta	NM_000576
CCNB1IP1	Cyclin B1 interacting protein1	NM_182852		***IL1RN/ IL1Ra***	Interleukin 1 receptor antagonist	NM_173842
CD177	CD177 molecule	NM_020406		***IL2***	Interleukin 2	NM_000586
CD274	CD274 molecule	NM_014143<br>NM_001267706		***IL7R***	Interleukin 7 receptor	NM_002185
CD3D	CD3d molecule	NM_000732<br>NM_001040651		***IP10/ CXCL10***	Interferon gamma induced protein 10	NM_001565
CD64	Fc gamma receptor type I	NM_000566		***LILRB2***	Leukocyte immunoglobulin like receptor B2	NM_001278403

CD74	CD74 molecule	NM_004355<br>NM_001025158<br>NM_001025159	***MDC1***	Mediator of DNA damage checkpoint 1	NM_014641
CIITA	Class II major histocompatibility complex transactivator	NM000246	***NFkB1***	Nuclear factor kappa B subunit 1	NM_003998<br>NM_001165412<br>NM_001319226
CTLA4	Cytotoxic T-lymphocyte associated protein 4	NM_005214<br>NM_001037631	***OAS2***	2'-5'-oligo adenylate synthetase 2	NM_016817
CX3CR1	C-X3-C motif chemokine receptor 1	NM_001337<br>NM_001171171<br>NM_001171172<br>NM_001171174	***RORγt/ RORc***	RAR related orphan receptor C	NM_001001523
FAS	Fas cell surface death receptor	NM_000043	***S100A9***	S100 calcium binding protein A9	NM_002965
FLT1/ VGFR1	Fms related tyrosine kinase 1/ The vascular endothelial growth factor	NM_002019<br>NM_001159920<br>NM_001160030<br>NM_001160031	***TBX21***	T-box transcription factor 21	NM_013351
GATA3	GATA binding protein 3	NM_001002295<br>NM_002051	***TDRD9***	Tudor domain containing 9	NM_153046
GNLY	Granulysin	NM_012483	***TIM3/ HAVCR2***	Hepatitis A virus cellular receptor 2	NM_032782
GSN	Gelsolin	NM_001127663	***TNFα***	Tumor necrosis factor alpha	NM_000594
HIF1α	Hypoxia inducible factor 1 subunit alpha	NM_001243084	***TREM1***	Triggering receptor expressed on myeloid cells1	NM_018643<br>NM_001242590<br>NM_001242589
IFNγ	Interferon gamma	NM_000619	***ZAP70***	Zeta chain of T cell receptor associated protein kinase 70	NM207519

Fig. S1 Kinetics of the absolute count of immune cells in response to LPS. **A.** The absolute counts of eosinophil and monocyte cells. **B.** The absolute count of neutrophil and lymphocyte cells. The absolute counts are expressed as 10^9 cells per liter blood, shown before (T0) and 4 hours after the *in vivo* LPS challenge (T4).

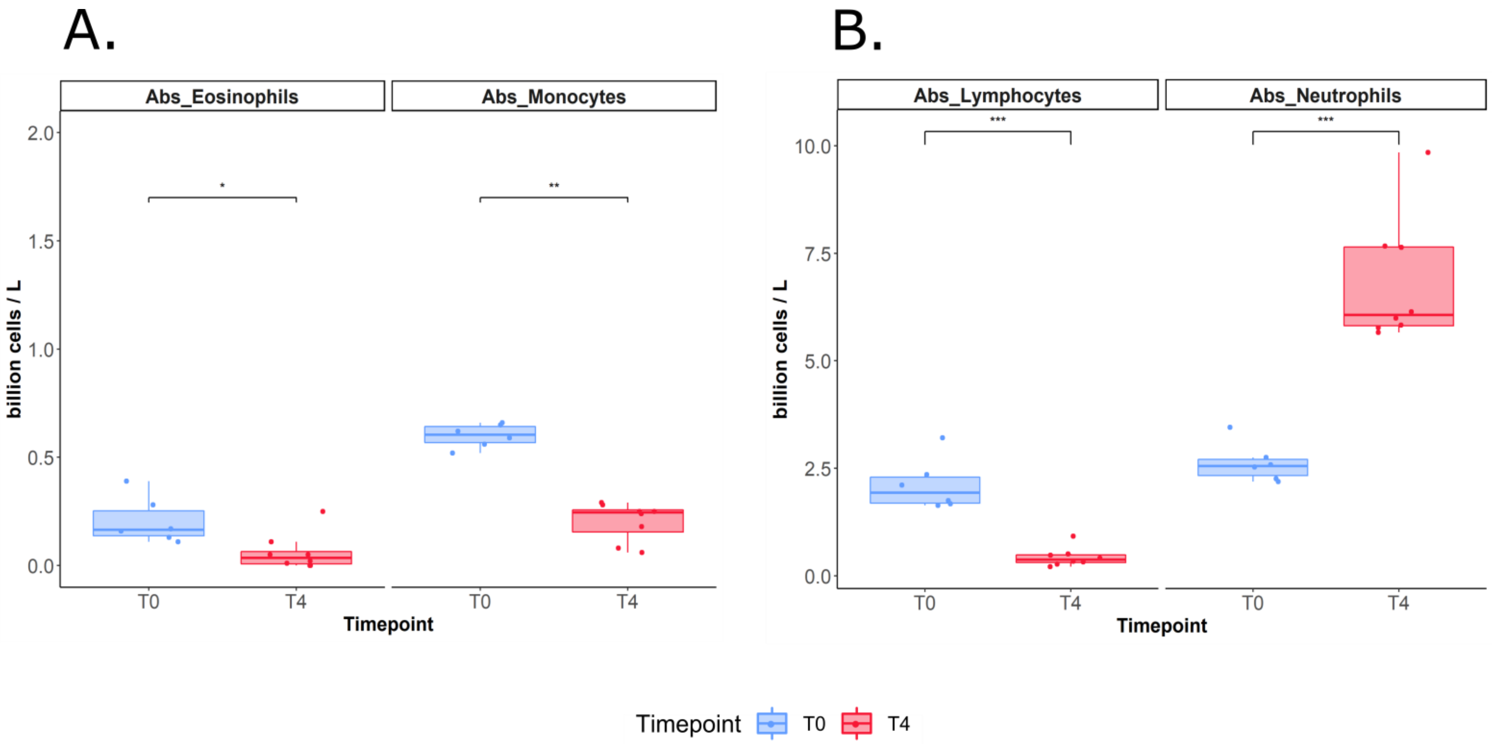

Fig. S2 Principal component analysis of the biological parameters and immune cell counts measured in the human *in vivo* endotoxemia model. The highest contributing factors to the LPS challenge are presented in dark red, and the colors degrade until the lowest contributing factors presented in green. Cos2 represents the quality of projection of each marker on the PCA plot which is expressed by the transparency of the arrows.

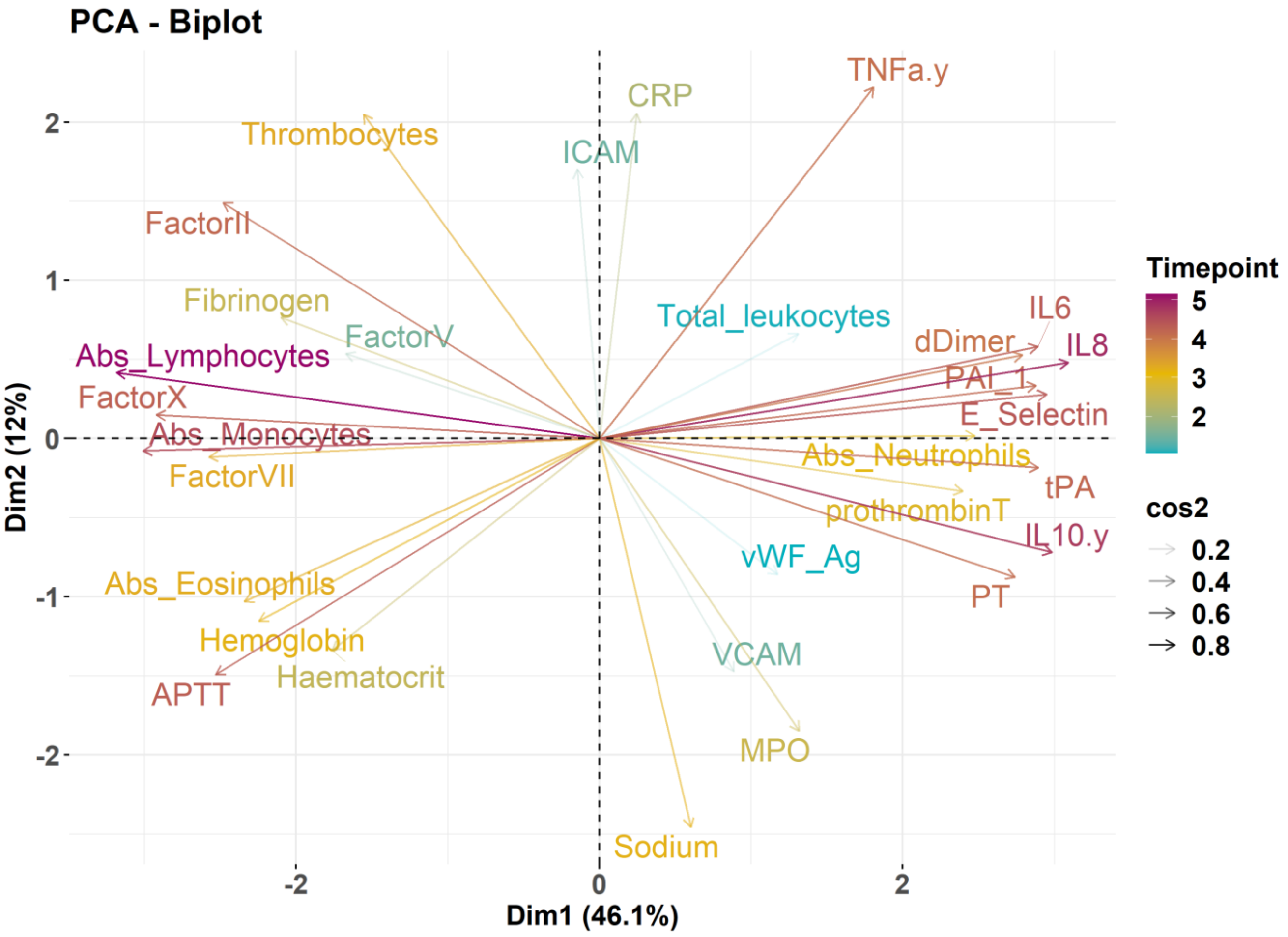

Fig. S3 Pearson correlations of IPP markers with the biological parameters and immune cell counts in human *in vivo* endotoxemia model. Correlations ≥ 0.7 Pearson coefficient are only shown and the cut-off of 0.7 was selected as an indicator of strong correlations. A. Markers that were upregulated 4 hours after the LPS challenge B. The downregulated markers after 4 hours of the LPS challenge.

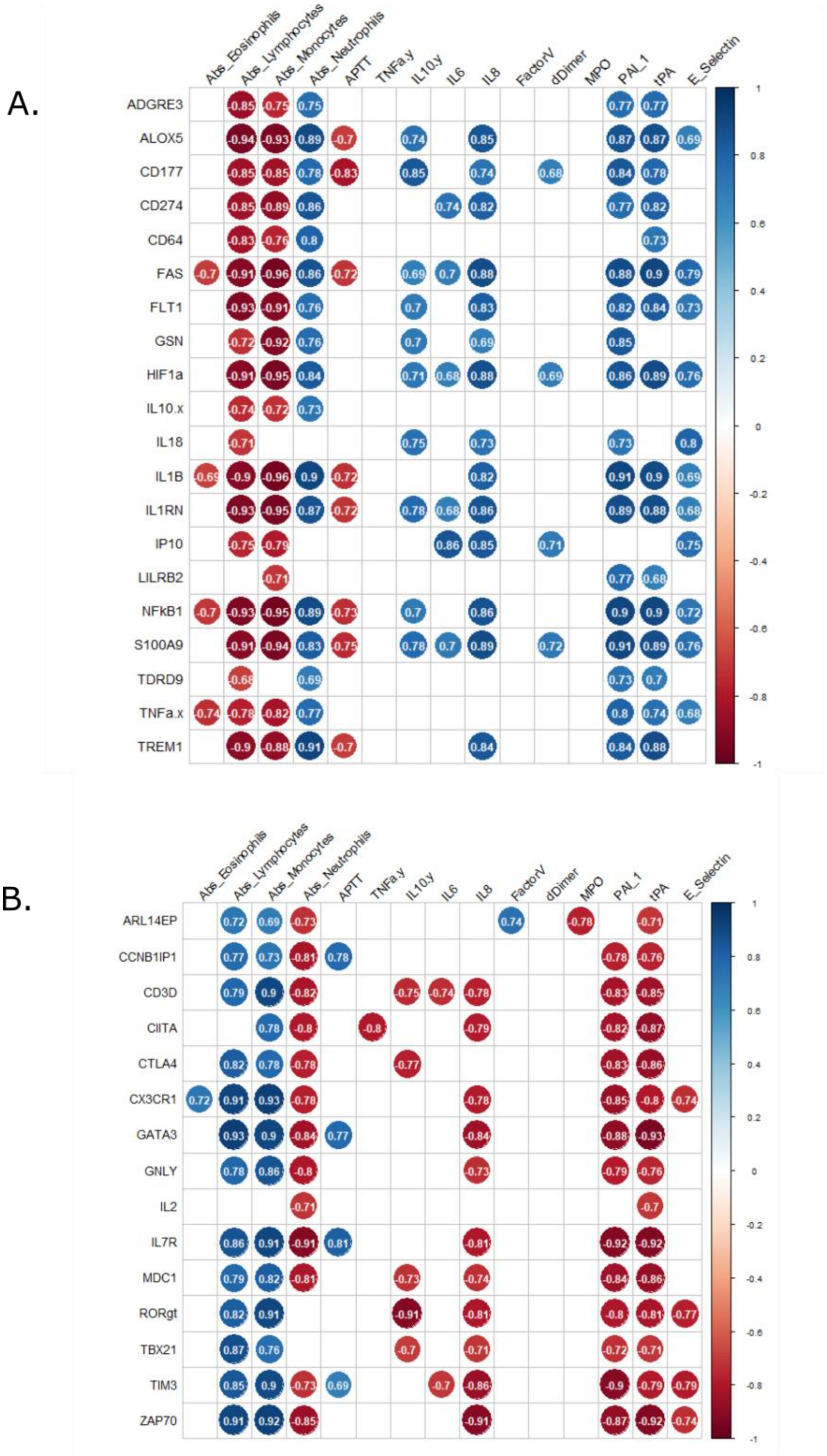

Fig. S4 Boxplots of various IPP markers corrected with the count of relevant immune cells. **A.** *CD177*, *CD64*, *S100A9* and *TREM1* adjusted to neutrophil count. **B.** *CD3D*, *GATA3*, *GNLY* and *TBX21* adjusted to lymphocyte count and **C.** CD74, CIITA, *CX3CR1* and *TNF-α* adjusted to monocyte count. The adjusted expressions were compared using paired Wilcoxon signed rank test (where NS: $p > 0.05$, *: $p < 0.05$, **: $p < 0.01$ and ***: $p < 0.001$). Two individuals observations are missing in all the immune cell counts reading at T0 only, and one observation is missing for *CD177* at T0.

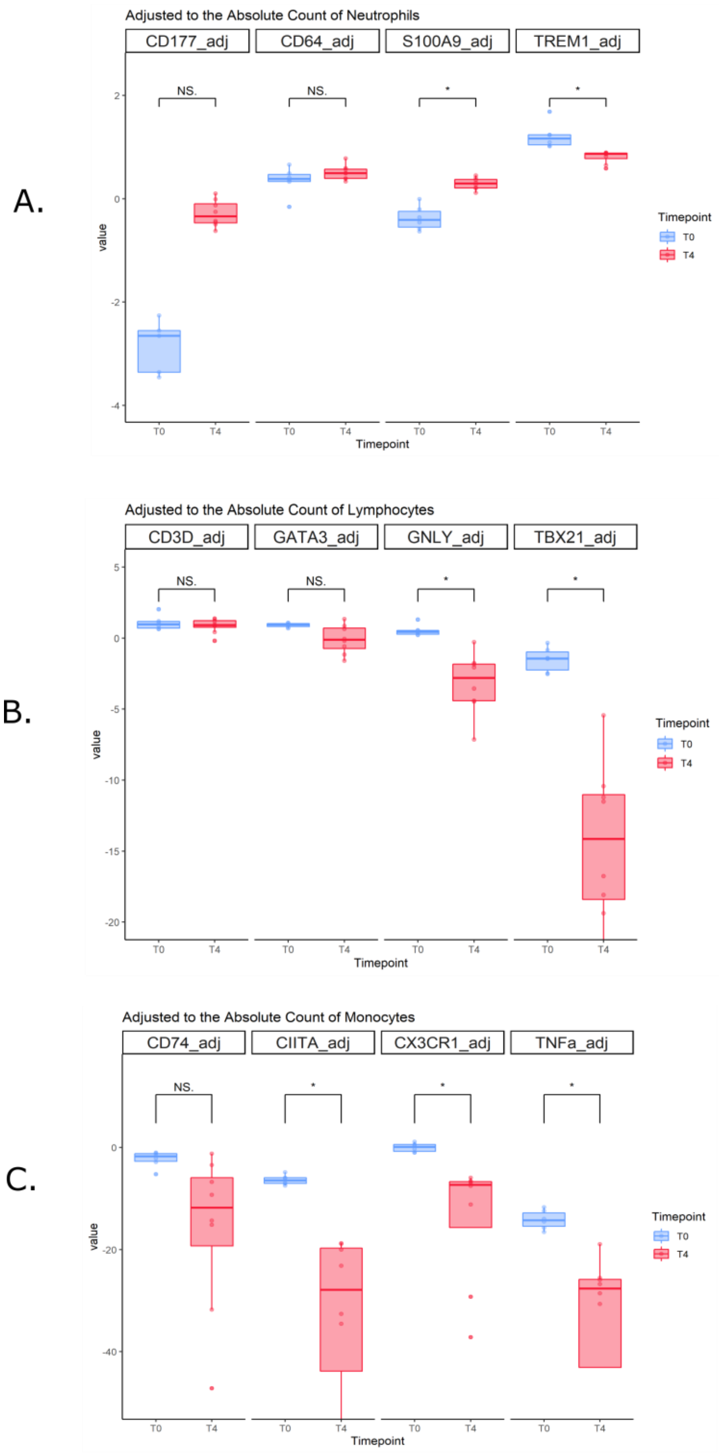

Supplement: Supplementary file 1 — Supplementary information. [file 41598_2020_66695_MOESM1_ESM.pdf]
